# Perceived Use Cases, Barriers, and Requirements for a Smart Health-Tracking Toilet Seat: Qualitative Focus Group Study

**DOI:** 10.2196/44850

**Published:** 2023-08-11

**Authors:** Sander Hermsen, Vera Verbiest, Marije Buijs, Eva Wentink

**Affiliations:** 1 OnePlanet Research Center Wageningen Netherlands; 2 Radboud University Nijmegen Netherlands

**Keywords:** digital health, internet of things, human factors, health tracking, device, automated, biomarker, personal health, personal hygiene, hygiene, data, privacy, innovation, mobile phone

## Abstract

**Background:**

Smart bathroom technology offers unrivaled opportunities for the automated measurement of a range of biomarkers and other data. Unfortunately, efforts in this area are mostly driven by a *technology push* rather than *market pull* approach, which decreases the chances of successful adoption. As yet, little is known about the use cases, barriers, and desires that potential users of smart bathrooms perceive.

**Objective:**

This study aimed to investigate how participants from the general population experience using a smart sensor-equipped toilet seat installed in their home. The study contributes to answering the following questions: What use cases do citizens see for this innovation? and What are the limitations and barriers to its everyday use that they see, including concerns regarding privacy, the lack of fit with everyday practices, and unmet expectations for user experience?

**Methods:**

Overall, 31 participants from 30 households participated in a study consisting of 3 (partially overlapping) stages: sensitizing, in which participants filled out questionnaires to trigger their thoughts about smart bathroom use and personal health; provotyping, in which participants received a gentle provocation in the form of a smart toilet seat, which they used for 2 weeks; and discussion, in which participants took part in a web-based focus group session to discuss their experiences.

**Results:**

Participants mostly found the everyday use of the toilet, including installation and dismantling when necessary, to be relatively easy and free of complications. Where complications occurred, participants mentioned issues related to the design of the prototype, technology, or mismatches with normal practices in using toilets and hygiene. A broad range of use cases were mentioned, ranging from signaling potentially detrimental health conditions or exacerbations of existing conditions to documenting physical data to measuring biomarkers to inform a diagnosis and behavioral change. Participants differed greatly in whether they let others use, or even know about, the seat. Ownership and control over their own data were essential for most participants.

**Conclusions:**

This study showed that participants felt that a smart toilet seat could be acceptable and effective, as long as it fits everyday practices concerning toilet use and hygiene. The range of potential uses for a smart toilet seat is broad, as long as privacy and control over disclosure and data are warranted.

## Introduction

### Background

With the rapid development of sensor technology and machine learning, novel opportunities for the unobtrusive and continuous detection of health issues have arisen. These opportunities have the potential to improve the prevention and treatment of debilitating health conditions through early detection and exacerbation signaling while also reducing patient burden by making invasive testing redundant. In theory, almost every object surrounding people in daily life could be transformed into a smart entity by equipping it with sensors, actuators, and algorithms for the automatic evaluation of generated data. One promising area where unobtrusive and continuous detection can lead to great health benefits is the toilet. First, the toilet is a location where nearly everybody spends time regularly. Second, the toilet offers unrivaled opportunities for the automated measurement of a range of biomarkers and other data. The consistency, color, and density of urine, for instance, could offer insights into water-loss dehydration [1], a condition that occurs in 20% to 30% of the older population [2]. Ketones in urine are useful for detecting type II diabetes [3,4], a condition that affects >500 million people worldwide, with prevalence expected to grow even further in the next 10 years [5]. Detecting albumin and creatinine in urine can shed light on kidney failure [6]. Urine and stool contain proteins and leukocytes, which can provide information on the prevalence of inflammatory bowel disease [7], which exceeds 0.3% of the population in North America, Oceania, and most of Europe [8]. Similarly, many people have, or are at risk of, debilitating conditions associated with high blood pressure, which can also be measured during a bathroom visit, for instance, through strain detection [9].

With such great potential for automatic, unobtrusive assessment of relevant biomarkers, it may be no surprise that there have been several recent initiatives to develop such a *smart* toilet (eg, the studies by Wang and Camilleri [10], Bhatia et al [11], Bae and Lee [12], and Balaceanu et al [13]). These initiatives are mostly driven by a *technology push* rather than *market pull* approach: scientific and technological innovations serve as the drivers of solutions to societal problems rather than direct demand from a customer or an envisioned target population [14,15]. Technology push approaches play a major role in innovation, both by providing solutions for gaps between the status quo and desired societal states and by enabling new modes of idea generation and selection [16]. However, this approach is not without risks. A limited connection to people’s goals and barriers often leads to nonimplementation [17]. Furthermore, when implemented, 80% of newly introduced inventions fail within 2 years [18].

A major factor determining the success of technology push–driven innovations is the consideration of the barriers and needs of potential end users [15,18], with unmet demands and needs known to significantly impact the sustained use of digital tracking devices [19]. Traditional ways to incorporate user needs and demands into the development process are user experience research, which evaluates users’ opinions regarding the esthetic, hedonic, affective, or experiential aspects of the use of a given technological prototype [20], and user-centered design, a methodology for placing users at the center of the development process from the early stages of designing system requirements to implementing and evaluating the product [21]. Although there is a definite value in having potential users of an innovation take part in the development process, this involvement is the most valuable when at the very conception of the innovation [22].

Recently, there have been new developments in early user involvement in scientific research under the guise of *extreme citizen science*, a participatory research approach in which citizens not only take part in gathering data but also codetermine the research agenda. Typically, when the term “citizen science” is used to describe a scientific work, it indicates that nonprofessional researchers gathered and occasionally processed data as part of the larger research endeavor. The widespread use of information and communication technology in general and ubiquitous computing in particular; the understanding that the public can supply free labor, skills, computing power, and even funding (crowdsourcing and crowdfunding); and the rising expectations of research funders for public engagement are all significant driving forces behind the recent growth in citizen involvement in research [23,24]. A relatively new development, however, is the development of extreme participatory approaches, in which citizens’ needs not only inform the development of an innovation but also determine the research questions that set the research agenda for the intervention development in the first place.

Unfortunately, how citizens can play this role often remains elusive. Turning everyday health challenges into research questions requires knowledge and skills that many people lack. Therefore, this study uses a citizen science approach based on participatory design methodology [25,26] to support citizens in capturing the potential use cases, user needs, and perceived barriers for smart sensor technologies in the bathroom. This methodology helps participants think about their situation and the ways in which technological innovations can or cannot support them in managing health conditions and living their everyday lives.

Although the discussion phase is exploratory and open to any input participants may provide, literature can already elucidate some of the themes that are likely to arise when thinking about, or trying out, smart bathroom technology innovations. First, the literature can shape one’s expectations of how smart bathroom innovations interact with everyday practices; these practices can be thought of as the interplay of practical knowledge, common understandings, rules, and material infrastructure that determines our expectations and behaviors at certain moments and places [27]. How the smart bathroom fits with people’s knowledge, common ideas and norms, and expectations surrounding toilet use determines the way in which it will be accepted, rejected, or even subverted for other use by future users [28]. This not only sheds light on the feasibility of the innovation but can also inform the design of future iterations of the innovation prototype. Second, literature on the use cases of technological innovations in everyday life shows that people have different uses for tracking technology, including directive tracking aimed at behavioral change; documentary tracking aimed at finding out more about oneself; diagnostic tracking aimed at answering questions about one’s health; tracking aimed at collecting rewards; and so-called fetishized tracking, that is, using technology out of love for the technology itself [29,30]. Third, the literature shows that sensing technology introduced in sensitive domains of everyday life triggers different privacy needs in different people [31]; some people are willing to use the technology out in the open and even use it to strengthen their social identity, whereas others are more reserved or hide the technology from others altogether—often referred to in the literature as on-stage use, off-stage use, and backstage use of technology, respectively [32]. Finally, data sharing needs, perceived barriers to sharing data, and privacy requirements differ between people [33], with some people being more trusting and willing to share data than others.

### Goal of This Study

This study, therefore, aimed to investigate how participants from the general population experience using a smart toilet seat equipped with sensors for body temperature, weight, electrocardiogram, bioimpedance, and photoplethysmography installed in their home. The study contributes to answering the following questions: What use cases do citizens see for this innovation? and What are the limitations and barriers to its use in everyday life that they see?

## Methods

### Overview

The aim of this study was to investigate how participants from the general population experience using a smart toilet seat installed in their home and what use cases they foresee for such a toilet. The study consisted of 3 stages: sensitizing, provotyping, and discussion. The sensitizing [34] stage aims to help participants think about different aspects of the innovation. To do so, participants generally read materials, watch film clips, keep diaries, or fill out questionnaires that help them notice aspects and form their thoughts. In this study, participants filled out questionnaires to trigger their thoughts about smart bathroom use and personal health. The *provotyping* stage, a combination of “provocation” and “prototyping” [35,36], lets participants work with prototypes, often with low fidelity, of the innovation as a safe, gentle provocation. This helps elicit tacit knowledge such as everyday practices, norms, cultural conventions, and taboos. In this study, the provotype participants used a smart toilet seat for 2 weeks. The third stage is *discussion*, in which scientists and citizens explore themes and solution spaces together, based on the insights gathered in the sensitizing and provotyping stages. The participants took part in a web-based focus group session to discuss their experiences. In this study, the sensitizing and provotyping stages mostly overlapped. The recordings of the focus group sessions were transcribed and analyzed using thematic qualitative analysis.

This study was part of a larger trial testing the efficacy of sensors installed in the toilet: electrocardiogram sensors, bioimpedance sensors, photoplethysmography sensors, weight sensors, and body temperature sensors. The trial tested whether the sensors delivered adequate data quality to inform measurements and predictions and whether the data from the sensors enabled distinction between the different users of the toilet. We could not guarantee that the quality of the data was sufficient to provide valid and reliable feedback on biomarkers to participants. Furthermore, the data provided by the sensors did not contribute to the answering of the research questions in this paper. Therefore, participants received no feedback from sensor data of any kind, nor was the analysis and reporting of the sensor data part of this paper.

### Participants

#### Overview

We aimed to include people from the general population, aged ≥16 years, and potentially interested in using a smart toilet. Participants were recruited from the province of Gelderland in the Netherlands and its neighboring regions owing to logistic restrictions in delivering and installing the toilet. To capture a potentially broad range of potential use cases, we aimed to include participants from all age groups, except children aged <16 years who could use the toilet as part of a participating household but could not actively participate and provide data. People weighing >100 kg were excluded from the study, as well as people with pacemakers and pregnant women, because the smart toilet prototype had not yet been tested for use with these groups. Because of the exploratory nature of the research, which aimed at generating use cases from a large populace and not specific groups, we added no further inclusion or exclusion criteria.

Because most potential participants lived in a household consisting of >1 person and the toilet seat collects data from every person using it, all members of the partaking households needed to give their consent to the collection of their physiological signals via the toilet seat. Therefore, we set up 2 levels of participation: active participation, in which the participant filled out all questionnaires and took part in the discussion session, and passive participation, in which the participant used the smart toilet but did not want their data to be used in the analysis. Passive participants did not fill out any questionnaires and did not take part in the discussion, and their physiological data were deleted after the measurement period. Only data from active participants were included in this study.

#### Recruitment

Participant recruitment took place through various publications in regional media, such as local newspapers and web-based news sites, and social media. Participants could indicate their interest by sending an email to the study coordinator, who then contacted them via email to inform them about the study procedure, aims, and time frame and share the consent form. If participants had any questions, the study coordinator answered them via email or telephone. If participants then agreed to take part, they filled out the consent form upon the delivery and installation of the smart toilet seat. Participants received no monetary or other remuneration for taking part.

#### Sample Size Considerations

In qualitative research, a priori sample size calculations are subject to conceptual debate and practical uncertainty. Saturation, that is, the moment when adding more data does not lead to new insights, is often seen as a criterion for the inclusion of more participants once the analysis has started. As a rule of thumb, 20 to 40 participants are usually considered sufficient to achieve saturation [37,38]. Given these considerations and the possibility of withdrawal, we aimed to recruit participants from 30 to 40 households for this study, with at least 1 participant per household. To ascertain a broad range of potential use cases, we aimed to recruit people from different age brackets, preferably >5 participants aged 16 to 30 years, >5 participants aged 31 to 45 years, >5 participants aged 46 to 65 years, and >5 participants aged >65 years.

### Ethical Considerations

This study was deemed exempt from ethics approval according to the Dutch Medical Research Involving Human Subjects Act (Wet Medisch Onderzoek) by the medical ethical committee of the Maxima Medical Center in Veldhoven, Netherlands (decision number N21.090). An extensive risk assessment was performed and did not reveal any risks exceeding the acceptable limits, and possible risks were mitigated as much as possible. This study fully adhered to the Declaration of Helsinki, 2013 amendment.

### Consent to Participate

All active and passive participants provided full written consent for their participation and the use of their data for scientific publishing and other dissemination purposes. Participants were briefed about the procedure and goal of the study and were aware that they could leave the trial at any point in time if they wished to do so without any consequences or obligation to give a reason.

### Procedure and Materials

#### Overview

Upon the confirmation of participation, the research team sent out an information leaflet with general information; the goal, procedure, and background of the research; eligibility criteria; privacy considerations; and procedures for withdrawal and consent forms. They then made an appointment to deliver the smart toilet seat to the home of the participants. During the visit, all participants, both active and passive, signed the informed consent forms. Consent for participants aged <18 years was provided by their parents.

#### Sensitizing Phase: Questionnaires

Shortly after the installation of the toilet seat, all active participants filled out a web-based questionnaire on their mental well-being, gut health, overall health, and expectations toward the smart bathroom. To do so, they received an email containing an invitation link to the questionnaire, which was delivered through a web-based questionnaire delivery service (Castor EDC) and filled out on the participants’ own laptop, tablet, or smartphone. After this, participants received an email link to a second questionnaire, also delivered through Castor EDC, with questions regarding the toilet installation process. During the 2-week use period, every evening at 7 PM, all active participants received an invitation to fill out a brief questionnaire via an ecological momentary assessment (EMA) app, which they had to install on their smartphone to participate in the study. To reduce their burden, participants were free to fill out or ignore the EMAs after filling out at least 4 of them during the 2-week study period to support the linking of sensor data to particular active users (not covered in this paper). The EMA questionnaires polled participants on toilet use but also contained 1 question each about general health, mood, and stress level and room to leave thoughts and questions about the smart toilet.

Finally, after the 2-week use period, active participants received an email invitation to a final questionnaire, which polled them about their experience using the smart toilet. This questionnaire contained questions from the Systemic Usability Scale (SUS) [39]. The SUS consists of 10 questions with a 5-item Likert scale ranging from “strongly disagree” to “strongly agree.” Because this scale has known limitations [40], additional items regarding the hedonic and pragmatic qualities of the prototype [41,42] were added. The hedonic quality of the prototype, which was measured on a 7-point Likert scale ranging from “strongly disagree” to “strongly agree,” corresponds to its valence and perceived usefulness, for example, its practicality, niceness, modernity, amusingness, credibility, ease of use, level of answering to needs, beauty, and robustness, and to disadvantages associated with its use, for example, intrusiveness, embarrassment, and nuisance. Pragmatic quality, measured on a similar 7-point scale, corresponds to the prototype’s perceived validity and reliability, for example, its exactness, level of detail, clarity, and credibility. Participants then filled out the Affinity for Technology Interaction (ATI) scale [43]. This questionnaire assesses a person’s tendency to actively engage in or avoid intensive technology interaction and consists of items measured on a 6-point Likert scale ranging from “completely disagree” to “completely agree.”

The main aim of the questionnaires was to help participants shape their thoughts; therefore, all questionnaire data were discarded, except for the general health and demographic information from the introductory questionnaire, open fields with thoughts and questions from the EMAs, and responses to questions on user experience and affinity to technology from the final questionnaire. All questionnaires are available in Multimedia Appendix 1.

#### Provotyping Phase: The Smart Toilet

Participants made use of an early prototype of a smart toilet seat currently under development at OnePlanet Research Center. The prototype was equipped with electrocardiogram and photoplethysmography sensors, a bioimpedance sensor, a thermometer, and weight sensors. These sensors provide a basic setup that affords the monitoring of the so-called vital signs [44]: blood pressure (electrocardiogram and photoplethysmography), pulse (electrocardiogram and photoplethysmography), body temperature (thermometer), respiration (bioimpedance), blood oxygen (electrocardiogram and photoplethysmography), and weight. Although there are multiple existing methods for measuring the vital signs, what sets the smart toilet apart is its ability to perform measurements automatically and unobtrusively a couple of times a day, which other methods lack, imposing a burden on the user. As stated in the introduction, this basic sensor suite could be expanded to include more sensors that analyze biomarkers in urine and stool and other sensors; however, time and budget constraints necessitate choosing the sensors that would have the most added value. This study was one of the activities performed by OnePlanet Research Center to identify such sensors.

**Figure 1:**
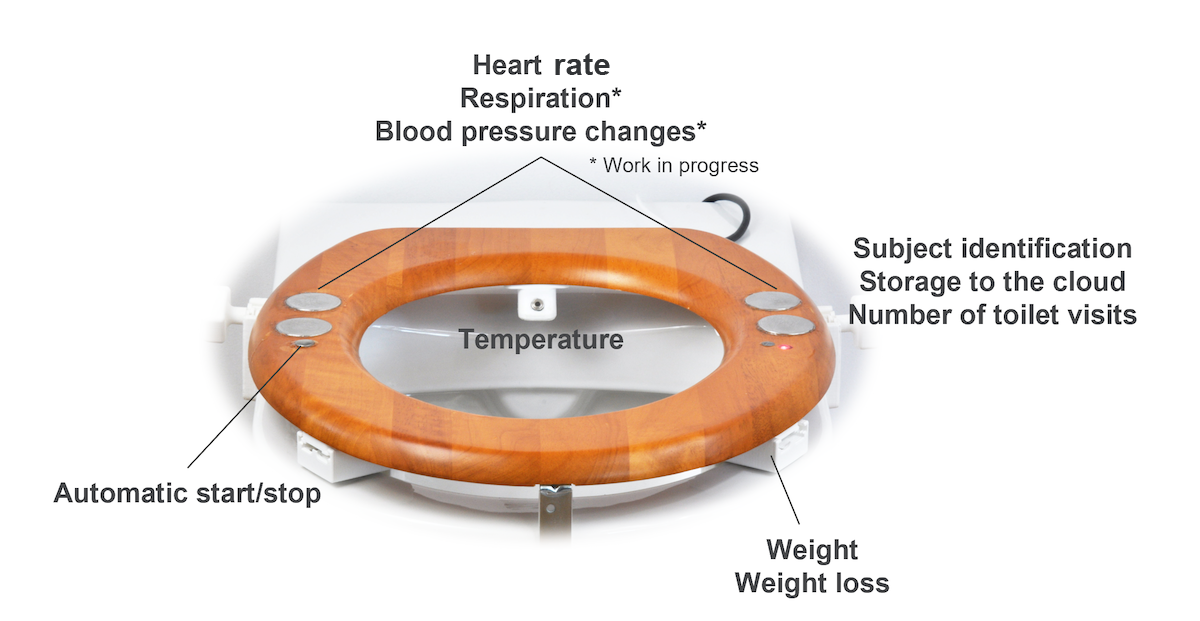
Smart toilet seat prototype. The seat has sensors for measuring physiological parameters and clamps for easy installation.

**Figure 2:**
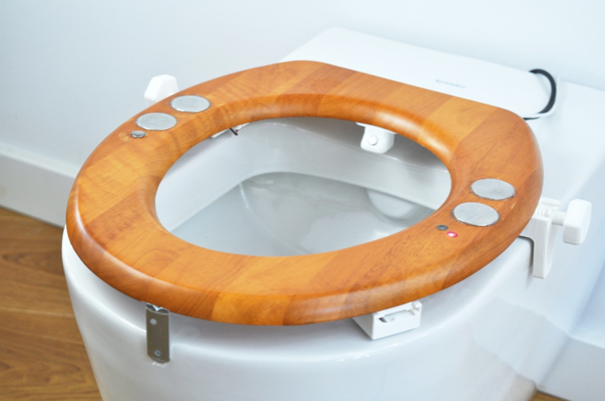
Photograph of the smart toilet seat installed on a regular toilet bowl.

**Figure 3:**
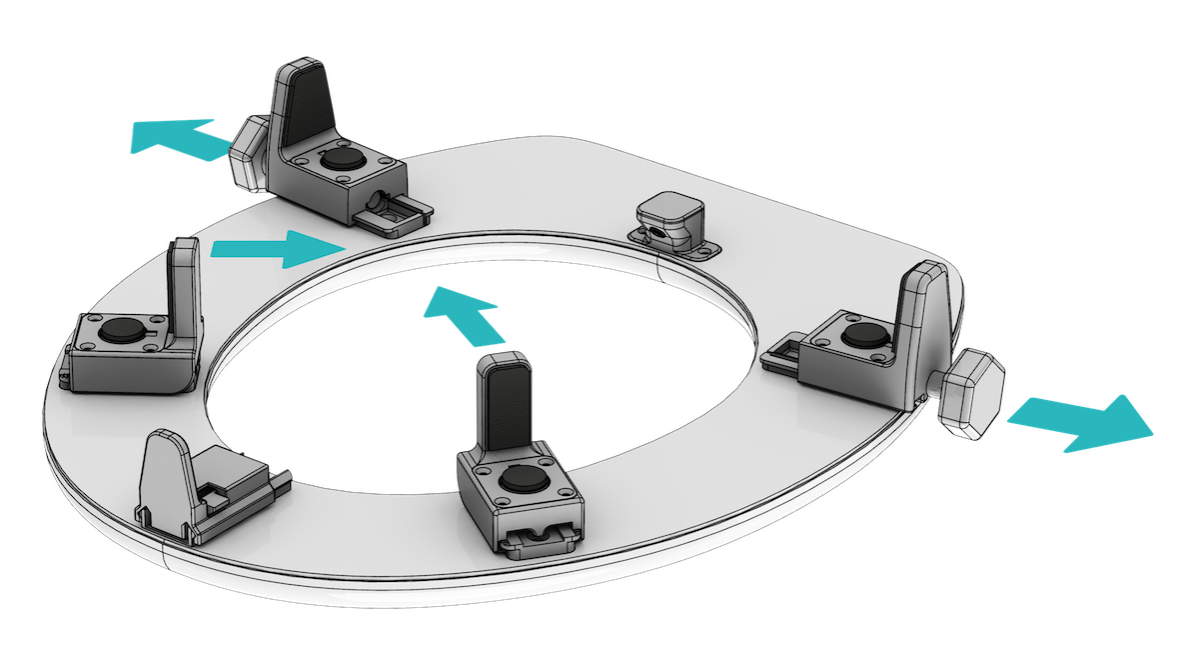
Installation clamps on the smart toilet seat. This illustration shows the bottom of the installation seat with the clamps needed for installation. Clamps 1 and 2 are being turned and 3 and 4 are being pushed, whereas clamp 5 requires pushing 2 buttons to move it. The arrows show the ways in which the clamps have to be placed before placing the smart toilet seat on the toilet bowl.

Participants installed the smart toilet seat (see Figure 1 for a schematic overview and Figure 2 for a photograph) with the use of an installation manual, by placing it on top of the regular toilet and fastening the clamps (see Figure 3 for an overview of the installation clamps). They then placed a transmitter device within 10 meters of the toilet seat but not necessarily inside the same room; the transmitter needs power from a mains socket, and these sockets are not always available in bathrooms. The transmitter automatically connected to the seat when powered up and sent all the collected data to a cloud-based secure storage. Activation of the sensors on the toilet could be identified through a red light of the photoplethysmography sensor that turned on when the seat came into contact with the skin. The connection of the seat with the transmitter box could be checked through a blue light on the transmitter. Before participants used the seat, one of the researchers checked whether the sensors produced data and whether the data were sent to secure servers.

Participants then used the toilet seat for 2 weeks as they normally would, with no additional action needed when visiting the toilet, except from sitting down for urinating, which may be uncommon for some male participants. In the information leaflet, participants could read that the toilet measured their heart rate, body temperature, breathing rate, and weight and that the toilet would transfer this information to a secure cloud storage facility to be able to determine the signal quality. Participants were aware that the data would not be used for any kind of diagnosis or comparison outside of determining the adequacy of signal quality and who was using the toilet; they were also aware that they would not receive feedback on their health or toilet use at any time. After the 2-week period, the seats were disassembled and picked up by the researchers.

#### Discussion Phase: Focus Group Sessions

In the week after completing the 2-week provotyping phase, all active participants took part in 1 of the 12 web-based focus group discussion sessions, which lasted 45 minutes to 1 hour. These sessions took place through web-based meeting platforms, either Microsoft Teams (Microsoft Corp) or Jitsi Meet (8x8, Inc). The aim was to have 3 to 6 participants and 2 researchers (from a group of 3: the first, second, and third authors of this manuscript) in each session. One researcher played the role of a discussion leader, and the other researcher played the role of an observer and supported the discussion leader where needed.

During the discussion sessions, each participant individually reacted to five discussion theses: (1) their thoughts on the sensitizer materials; (2) their overall experience during the study, such as during the installation, removal, use, and cleaning of the seat; (3) their perceived use cases for the smart toilet seat; (4) how they felt about others knowing about their having and using a smart toilet seat; and (5) their opinion on smart toilet seat data privacy. After each participant gave their opinion on a thesis, all other participants had the opportunity to freely react to what they had heard. Every session was recorded; recording was started only after the confirmation of consent from each participant. The session recordings were transcribed and deleted directly after checking the transcription.

To conclude the project, participants received an extensive briefing of the study results, which included the main insights described in this manuscript. The briefing contained no feedback on personal physiological data.

### Analysis

#### Sensitizing and Provotyping Phases

Because the sensitizing questionnaires only served to inform participants’ thoughts about their everyday situation and the use of the smart toilet, we discarded all the data from the sensitizing questionnaires, except for some demographic data (age, gender, and general health status) and the responses to the usability questionnaires (SUS and questionnaires covering hedonic, pragmatic, and efficacy aspects) and the ATI scale. For the user experience questionnaires and ATI scale, descriptive results were calculated: means, medians, and SDs. From the EMA questionnaires, we listed and grouped the open entries with thoughts people had about the smart toilet. No further data from the provocation phase, such as the sensor data, were analyzed in this study. The SUS score was calculated using the following formula: *SUS score = ([score of items 1+3+5+7+9] − 5) + (25 − [score of items 2+4+6+8+10]) × 2.5*, which gives a score ranging from 0 to 100. The hedonic quality and pragmatic quality were calculated by taking the mean of the corresponding questionnaire items. The ATI score was calculated by taking the mean of the 9 items and comparing it with the average score of a similar population [43].

#### Discussion Phase

Two researchers (MB and SH) manually transcribed the recordings of the discussion sessions. They anonymized the transcript by removing personal information. All transcripts were then read into a qualitative analysis software [45] and analyzed using a method based on inductive thematic analysis [46,47]. Following this approach [47,48], 2 researchers (MB and SH) first performed a primary analysis of 2 session transcripts individually, from which an initial coding scheme emerged, and then compared their coding to ascertain similar interpretations. They further applied inductive coding to identify themes and patterns in the data not yet covered in the coding scheme and then applied the updated coding scheme to the first 5 transcripts. A further iteration of the analysis then took place to ascertain confidence in the coding. The coding scheme was then modified to better reflect emergent themes, and all relevant text segments were coded. This step was repeated until no more issues arose.

### Reflexivity

In any research where the researcher attempts to make sense of participants’ experiences, there is a potential risk of researcher bias [49]. To improve the integrity and credibility of qualitative research, researchers must assess how intersubjective components affect data collection and analysis. An instrument for this examination is reflexivity, which refers to researchers’ explicit, self-aware appraisal of their own roles [49,50].

The host institute of the study reported in this manuscript, OnePlanet Research Center, researches potential innovations in health and sustainability using sensor technology and artificial intelligence. One of its research programs is on gut health, in which the smart bathroom is an important part. The end goal of the program is an integrated suite of sensors that informs a personal digital twin model that can be used for signaling, measuring, and preventing a range of health conditions.

SH is the principal behavioral scientist at OnePlanet, leading the human factors research at the center. His work focuses on the acceptability, usability, and efficacy of technological innovations for supporting people in healthy living.

VV is a biomedical field engineer at OnePlanet and is responsible for the design and performance of feasibility and efficacy studies.

MB worked on the research project as partial fulfillment of her Master’s Degree in Science, Management and Innovation at Radboud University Nijmegen.

EW is the principal investigator of the smart bathroom program; she leads all scientific and developmental activities for the smart toilet seat and other innovations.

## Results

### Participants

#### Overview

In total, 37 households took part in the study. Of these 37 households, 11 (30%) had >1 active participant, rendering a total of 49 active participants. During the trial, 1 (3%) household containing 1 (2%) participant quit; their data were discarded from the analysis. Of the remaining 48 (98%) active participants, 24 (50%) indicated their sex as male, and 24 (50%) indicated their sex as female. Overall, 28 (58%) participants did not report having any chronic health issues, 2 (4%) participants reported having diabetes, 3 (6%) participants reported having heart problems, 4 (8%) participants reported having asthma or chronic obstructive pulmonary disease, and 8 (17%) participants reported having arthritis. The chronic illnesses mentioned once were bipolar depression, celiac disease, hypertension, heart valve leakage, restless bowel syndrome, and ulcerative colitis. Participants were, on average, aged 62 (SD 13.97; median 68; range 28-84) years.

#### Adjustments to Protocol

Unfortunately, we failed to recruit participants from every age bracket as planned. To be precise, only 2 (4%) participants were from the 18-30 years age bracket. However, all other age brackets had >5 (10%) participants as planned. Although the research team put much effort into planning discussion sessions such that they accommodated all participants, it turned out to be impossible to accommodate all the participants because of work schedules, illnesses (especially COVID-19), late cancellations, and the limited availability of participants in the same time frame. Moreover, 2 (4%) participants had to leave the focus group discussion within 10 minutes; they were excluded from the analysis because they did not have the opportunity to contribute to the conversation. Therefore, of the initial 48 active participants from 36 households, 31 (65%) participants from 30 (83%) households completed a focus group session.

### Sensitizing and Provotyping Phases

#### Adjustments to Protocol

Participants were instructed to use the smart toilet seat for 2 weeks continuously; however, 6 (12%) participants were absent for several days and continued to use the seat after returning home, prolonging their provotyping period by the number of days they missed (up to 1 week in 3 cases). One seat needed to be replaced owing to malfunctioning 4 days into the trial; the provotyping period of the corresponding participant was not prolonged. Two (4%) participants filled out their final questionnaire on paper printouts.

#### User Experience Questionnaires and ATI

The average SUS score was 77.92 (SD 12.81; range 74.20-81.64; 48/48, 100%). This shows acceptable usability and corresponds to the (traditional US) school grading scale of C [39]. To further determine user experience, we also calculated the hedonic quality, which had an average of 4.83 (SD 1.32; range 4.56-5.10; 47/48, 98%) on a scale ranging from 1 to 7. The pragmatic quality was, on average, 4.37 (SD 1.88; range 3.98-4.75; 48/48, 100%). On the ATI, participants scored an average of 3.63 (SD 1.08; range 1.00-5.89; 48/48, 100%), which is similar to a comparison population average of 3.5 [43].

#### EMA Open Questions

Participants filled out a total of 293 EMAs, with an average of 7.15 (SD 3.95; range 2-18) EMAs per participant. In 165 (56.3%) EMAs, participants did not leave any text in the open remark field, whereas in 128 (43.7%) EMAs, they did, amounting to an average of 3.12 (SD 2.46; range 0-13) EMAs per participant. An overview of the categorization of these remarks is provided in Table 1.

**Table 1:** Remarks from the open input field of the EMA^a^ questionnaires.

Remark	EMA event (n=293), n (%)	Example
No remarks entered	165 (65.3)	N/A^b^
Curiosity and compliments	17 (5.8)	“Excited about starting the research”
Ideas for use cases	15 (5.1)	“Could the toilet detect I tested positive for COVID-19?”
How does the smart toilet work?	13 (4.4)	“Does the toilet ‘know’ I drank a lot of coffee today?”
I would like feedback on my data	12 (4.1)	“I would like a display next to the toilet that shows my heart rate and temperature”
User experience—smart toilet construction	12 (4.1)	“The fastening of the toilet seat is unstable”
Does the smart toilet work?	8 (2.7)	“I hope the signal comes through; I can’t tell if it works”
Using the toilet is now automatic or I already forgot about it	8 (2.7)	“I just sit down and don’t think about it anymore”
Doubts about usefulness; no fun	7 (2.4)	“Will this study produce results?”
User experience—hygiene	6 (2)	“I find it difficult to clean the toilet with the seat”
Speaking to or reactions from others about the toilet	6 (2)	“I talked to my son and his friends about the toilet, and they are intrigued”
User experience—comfort and everyday use	4 (1.4)	“The seat is cold”
Am I doing this right?	3 (1)	“Do I sit at the right spot? Or not far back enough?”
Questions about the research	3 (1)	“Does it matter for the measurements that I’m taking medicine?”

^a^EMA: ecological momentary assessment.

^b^N/A: not applicable.

### Discussion Phase

#### Adjustments to Protocol

Because of the aforementioned difficulties in planning discussion sessions such that they accommodated all participants, the aim to have focus groups with 4 to 6 people was met for only 19 (61%) of the 31 participants; 10 participants (32%) took part in a focus group with 2 to 3 other participants. The remaining 2 (6%) participants were unable to take part in any of the proposed dates for sessions or expressed a strong preference not to join other participants, which led to these 2 participants being interviewed on their own.

#### Theme: Everyday Use and User Experience

Approximately half (16/31, 52%) of the participants considered the everyday use of the toilet, including its installation and dismantling when necessary, to be easy and free of complications. A total of 6 (19%) participants mentioned removing the toilet seat for cleaning; 9 (29%) other participants mentioned not removing the toilet seat at all:

I hardly noticed the difference with my own regular toilet seat.

P623

I cleaned it once, no wait, twice, and I removed the seat to do so. Well, that was easy enough.

P280

So I just left it on for the whole two weeks; I felt that that would improve the chance that everything would keep working as it should.

P265

However, most participants (including some who found the everyday use of the toilet uncomplicated) mentioned issues with using the smart toilet seat related to the design of the prototype, the technology, or mismatches with normal practices in using toilets. First, many participants commented on the design of the prototype, with vulnerability, especially of the clamps connecting the seat to the toilet bowl, being the main issue mentioned by 14 (45%) participants:

If you don’t sit on it correctly, it wobbles a bit.

P283

Well, using it was not hard, but when you undo the seat, what with all the wires and sensors, if I don’t pay attention and yank too hard, it might well fall off the toilet and everything stops working. So I just left it there.

P265

A total of 3 (10%) participants felt that the seat, which was a bit higher than a regular toilet seat because of the clamps and weight sensors, made the toilet too high for them:

I talked to [the researcher] about this, whether my feet could still reach the ground, so we tested that. And it turned out it was way too high, but that wasn’t a problem for the 14 days.

P506

Furthermore, 8 (26%) participants disliked the color and design of the seat, often citing a mismatch with the overall design of their bathroom:

Well, the color of the seat, I think brown is a nasty color. It does go well with excrement though.

P850

I did think that the brown color...My bathroom is all white and blue. I was glad I had my own toilet seat back afterwards.

P580

Second, the sensor technology in the seat raised questions among some participants. A total of 7 (23%) participants mentioned being intrigued by the red light of the temperature sensor, and for 7 (23%) more participants, this light led to a feeling of being observed:

The red light intrigued me. Sometimes it was on when I got up, and sometimes it wasn’t. Maybe it was not constantly measuring? Or only measuring for 30 seconds? I had no idea.

P166

It’s not a huge issue, but the red light did trigger a feeling of, well, red means something is wrong.

P781

Later on, when I had a look at all the sensors, I was wondering which sensor was which, and thought that it could be a camera. I thought that went a bit far, but oh well, it’s all in the game.

P471

Third, participants reported issues related to a mismatch with everyday habits and practices of toilet use. When the toilet did not match their expectations, this affected their acceptance of the toilet. Hygiene and expectations related to cleaning were the most important issues. All 31 participants mentioned cleaning in one way or another. A total of 15 (18%) participants said that cleaning the seat was difficult because of the clamps, immovability of the seat, and wires. In addition, 14 (45%) other participants mentioned cleaning but also said that they experienced no difficulty, and 2 (6%) participants admitted that they did not clean the toilet at all (and left that task to their spouses):

A normal toilet seat, you can easily put it up and clean the bottom side. This one needs to be taken off entirely.

P515

I noticed that our cleaning person was anxious to remove the seat, so I told them it was okay to just clean between the clamps for two weeks. But I noticed they were worried about that.

P781

Also mentioned often (11, 35%, participants) was the fact that the smart toilet seat cannot be put up, which means that all users, regardless of their biology, are expected to urinate sitting down:

I have had some gentlemen visiting, my neighbour and his son. And I tried to talk them into using the toilet as well. But when I said the seat does not go up, they fled!

P400

As a man, I’m not used to sitting down to urinate, and I found that quite troublesome, especially the first few days.

P768

Moreover, 2 (6%) participants mentioned that the seat lacked a cover, 1 (3%) participant did not dare use her bidet owing to the fear that it might affect the electronics, and 5 (16%) participants talked about how their “irregular” behavior affected measurements: fidgeting; sitting on the very front of the seat only; sitting on their underpants or bathrobe; and, in 1 case, changing clothes while on the toilet:

I don’t always sit still on the toilet. In the morning, I already take off my pyjama bottoms, and in the evening my trousers, that sort of thing. At one time I started wondering whether that affects the measurements...

P026

#### Theme: Perceived Use Cases

A central aim of the study was to find out what use cases potential users would have for the smart toilet. Participants mentioned five categories of use cases (in the order of number of participants mentioning the category): (1) signaling potentially detrimental health conditions or exacerbations of existing conditions; (2) documenting physical data to find out more about oneself; (3) measuring biomarkers to inform a diagnosis; (4) using the smart bathroom for personal science: measuring the results of experiments in lifestyle and nutrition; and (5) tracking biomarkers to inform and trigger behavioral change. No participant mentioned fetishized tracking, that is, tracking out of interest in technology use.

Most participants (25/31, 81%) saw signaling potentially detrimental changes in their health, an early warning system, as a major use case for the toilet seat. This signaling is passive, with measurements occurring in the background. In use cases involving signaling, participants wanted to receive feedback only when there is a need for action:

Someone I know has a heart condition, a leaky valve. She’s ailing a bit but what can you do; I think this would be a solution for her. The seat could notify her in time when her heart condition deteriorates.

P400

I think the benefit of the seat is that you sit on it regularly, so you get regular measurements and feedback, for instance of blood pressure. That would be important to me. If there’s an outlier, I know I need to do something about it.

P245

Colon cancer is a real silent killer. Once you have complaints, it’s often too late. If complaints come suddenly, then you notice, but if it comes gradually over a long time, you just don’t realise. And the seat could notice these incremental changes, for instance in how often you need to go. Then you could get a warning that it would be smart to have a colon examination done.

P283

Overall, 13 (42%) participants mentioned use cases related to signaling critical values associated with their current health conditions, such as cardiovascular disease, diabetes, and gut conditions:

I have ulcerative colitis, which is an inflammation of the gut, and maybe the seat could measure inflammation values in the excrement. And if they are at a high level, the seat could notify me and tell me to make an appointment at the hospital.

P843

I don’t visit my GP all the time, so it might be that when the GP finds out my blood pressure is too high, it may have been like that for a long period without you knowing. It would be great if I could get a signal that something could be amiss.

P850

Furthermore, 10 (32%) participants mentioned that signaling could also involve the automatic transfer of relevant information to care professionals:

I would hope that if I would need to see a doctor regularly, that the seat would limit the number of times I have to go there. If it would simply send through the data if something were off, and the doctors could then see that values have changed and we need to act, that would be beneficial.

P471

I am a cardiovascular patient, even if you cannot tell. And I regularly need to check blood pressure, or fever, or my heart rate. The seat could measure all that for my cardiologist and myself.

P768

A total of 5 (16%) participants saw uses for the seat as a personal alarm system for older adults living alone:

I have an acquaintance who is seventy years old, and he had a stroke. It was a week before anybody noticed and the police had to break the door. He’s now in the hospital in a serious state. If he had had a smart toilet, the seat could have notified other people that he wasn’t using the toilet anymore...If you’re not going to the toilet anymore, there is something wrong. Maybe you are on holiday, but what if you are just lying there with a stroke?

P515

Second, participants mentioned documenting their physical state as a major use case for the smart toilet seat; 19 (61%) participants mentioned documenting use cases, that is, registering physiological data to get to know oneself and one’s bodily processes:

I would love to see my own data, to find out what daily rhythms I have. For instance, how long it takes for me to go to the toilet after I have eaten, how fast my digestion is. I would want my data to be available to me to learn such things.

P166

I would be very interested in sugar content and salt content of my urine. That would give me valuable information about my health.

P781

Third, 13 (42%) participants mentioned use cases in which the seat can be used for diagnosing health conditions, such as type II diabetes or kidney failure:

I work at a medical laboratory, and we do a lot of urine sampling. The seat would be great to replace a burdensome examination, where people need to collect urine for 24 hours. We could do the first sample in the lab and let the toilet measure the rest...This would be great for diagnosing kidney patients, to check if they produce enough urine.

P026

What if you could determine if someone has diabetes or prediabetes? If you catch that in time, that could lead to less complications in the future.

P450

Fourth, 6 (19%) participants would want to use the smart toilet seat for so-called personal science, that is, doing small experiments to find out what affects one’s health:

I have high blood pressure, and I am trying to find a good balance in salt intake. And I would like to know how fast changes in salt intake affect my blood pressure. I just want to check those data every day.

P280

I stopped eating yoghurt and cruesli after dinner, because my glucose went way up right after that and that affected my sleep quality...I find those trends on a micro-level very interesting. We think we eat healthily but often we don’t always. So I would want to use it for a while to experiment with my diet.

P781

Fifth, 3 (10%) participants saw use cases involving directive tracking, that is, tracking data to inform behavioral change and habit formation:

Well, I think many people do not drink enough, so waste products cannot leave the body effectively and your urine gets very dark. If the seat could give people feedback on that, and tell them to drink more, that would be good. Also, many people have obstipation, and you can solve that for a great part to eat more fibers and drink more. That’s an easy solution, and if your toilet can tell you that, that would help.

P588

The toilet seat could measure how much protein a person should ingest. Now, we cannot always measure that, so we use a formula that does not always fit the person.

P450

**Figure 4:**
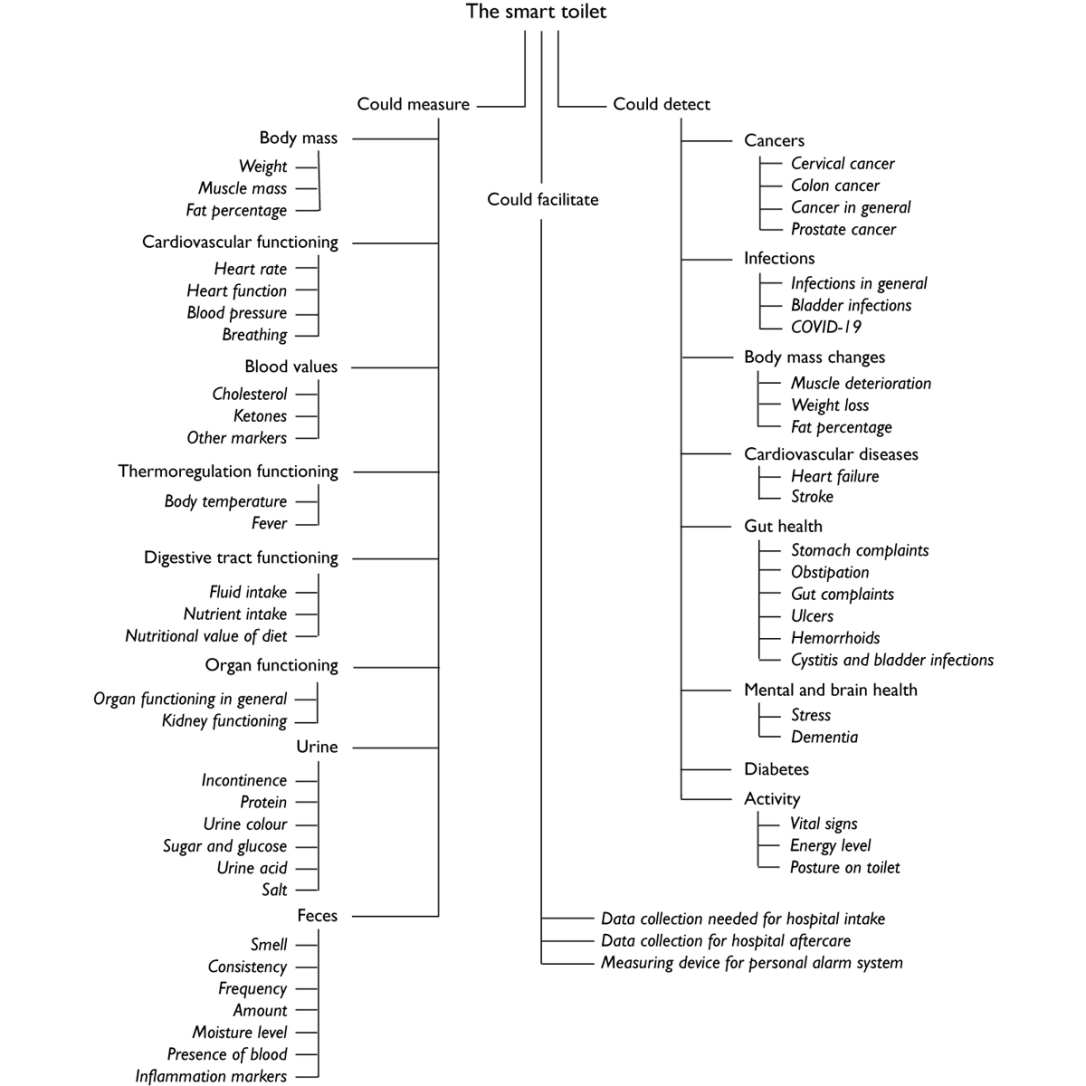
Overview of the potential use cases of the smart toilet.

The participants mentioned a broad range of conditions that they would like to assess using the smart toilet seat. These use cases ranged from being very vague (“telling me if something is off with my health”) to very specific (assessing salt and glucose levels in urine). A complete overview of the conditions mentioned by the participants is shown in Figure 4.

Other benefits of the smart toilet mentioned by the participants included a relief from burden for themselves and for their health care professionals, cost cutting, better targeted diagnosis, and better care in the period toward or after hospital care:

I work in the hospital as a dietician. To us, patient weight is very important. Especially older people don’t weigh themselves or have no scales at home. A smart toilet seat could alleviate the work of our nurses because they are under so much pressure, they cannot always weigh our patients. And that limits my work and my advice.

P450

Well, if you need regular check-ups in the hospital, they might arrange matters using the smart seat. Saves you a trip to the hospital.

P960

I have diabetes and would like constant monitoring to replace the finger-prick tests. And while we are at it, cholesterol as well.

P580

Finally, 17 (55%) participants mentioned concerns or doubts regarding the added value or efficacy of the smart toilet. A total of 13 (42%) participants thought that the smart seat had no added value when compared with existing measurement methods. Moreover, 5 (16%) participants mentioned the ways in which feedback on physiological data can have negative consequences, for instance, leading to heightened stress levels. The feasibility of measurements using the smart toilet seat was doubted by 4 (13%) participants, and 1 (3%) participant thought that health practitioners had no capacity to process the data that the smart toilet seat would generate:

I get nervous when I see a white coat, so my blood pressure rises when I know it gets measured. If I would get feedback from the toilet seat, that would probably make me nervous as well.

P506

What does the toilet have what other devices do not have? You can measure just about anything with a smart watch these days.

P561

I think that general practitioners aren’t happy when they get all sorts of data that they did not ask for. That will be a very difficult process to manage.

P841

And you know that if you want to measure blood pressure, you need to sit quietly and not move about. That is not easy on the toilet seat. The moment you sit there, you are already exerting yourself and that is going to influence the measurement. So I don’t know.

P768

#### Theme: Privacy and Data Sharing

An important theme, mentioned by all participants, was sharing the experience of using the smart toilet seat with others. Participants differed greatly in whether they let others use, or even know about, the seat. A total of 28 (90%) participants talked about experiences of engaging in social interactions regarding the toilet seat (on-stage use), whereas 13 (35%) participants talked about experiences of avoiding social interactions regarding the toilet seat (backstage use). Overall, 17 (55%) participants mentioned only positive sharing experiences; 4 (13%) participants mentioned only avoidance experiences; and 10 (32%) participants mentioned both categories of experiences, embracing social interactions regarding the toilet seat in some situations and avoiding them in others (off-stage use):

My neighbour and some other visitors, I led them to my upstairs bathroom, and told them ‘Have a look, will you?’ Another friend, I wanted to show her the smart seat, but she thought it would not suit her husband. If she were alone, she would want to try it at home as well. And I asked her whether she wanted to see the seat, but she didn’t.

P400

Well, it always led to conversations, right? Especially if there were young people who needed to use the toilet. I did warn them in advance, told them not to be scared. But everyone thought it was interesting and had all kinds of questions. I just told them it was something new, and maybe they would have something to do with it in the future.

P040

It’s nobody’s business. We don’t mind, but you don’t need to discuss toilet seats with your guests. Not because of etiquette or anything, but you just don’t.

P214

The participants who interacted with others regarding the seat did so on different levels. Overall, 16 (52%) participants mentioned talking about the smart seat with others, 6 (19%) participants mentioned showing the smart seat to visitors, and 10 (32%) participants mentioned letting other people use the smart seat. A total of 15 (48%) participants mentioned receiving positive responses when interacting with others about the smart seat, 4 (13%) participants mentioned receiving negative responses from others, and 4 (13%) participants mentioned receiving both positive and negative responses. With regard to positive responses, 13 (42%) participants mentioned others showing interest, 7 (23%) mentioned others having questions, 3 (10%) participants mentioned others being surprised, 3 (10%) participants mentioned others showing acceptance, and 1 (3%) participant mentioned that a visitor wanted to take part in the study as well and try the seat at home:

I talked quite a bit about it while walking the dog. I run in to a lot of dog owners and we chat, and then I would talk about the seat. People are very interested; they like to hear about it.

P471

It does evoke questions, you know. That makes sense, because suddenly there is an extra seat with a red light. So I can imagine people wonder. But that was not an issue, it just took some explaining with some people.

P500

Participants who did not interact with others regarding the smart seat had different strategies to avoid interaction. A total of 11 (35%) participants mentioned having toilets on different floors in their house and installing the smart seat in their upstairs bathroom so that visitors could use the downstairs toilet and not see the smart seat, 4 (13%) participants explicitly expressed not mentioning the smart seat to others, 2 (6%) participants took off the smart seat whenever people visited, and 1 (3%) participant mentioned the fact that nobody visited them during the 2-week period:

I did that on purpose [installing the toilet in the upstairs bathroom]. I reckoned that if I have visitors, I don’t want those people on that seat. That will just give rise to questions and remarks. So I just skirted around the issue; I thought let’s not have that.

P623

We have the privilege of having two downstairs toilets. I just used the one, and my wife used the other. But we did think about what the consequences would be if we did not have that; if we had visitors, we would have had to take the seat off and explain all kinds of things, and we would not want that.

P721

When it comes to sharing their data, all but 2 (6%) participants had reservations. A total of 15 (48%) participants explicitly mentioned that they want ownership of their data so that they can control who can see or use the data; 4 (13%) participants would only want to share secondary, derived data, such as blood pressure averages and trends, and not the raw data; 5 (16%) participants would share the data but only when anonymity can be guaranteed; and 12 (39%) participants said that they are suspicious or worried about data sharing:

What is important to me is that I have the say over the data. I want to decide who I share data with. Whether that’s my GP, or my neighbour to compare our physiology.

P166

Personally, I think online privacy is a false sense of security. Especially with smartphones. Every click you make gets measured by algorithms and sold to third parties.

P561

I am a bit scared that insurance companies and the like will draw all kinds of conclusions from the data.

P280

Among the 22 participants who would share their data, all but 2 (9%) participants would limit accessibility. A total of 18 (82%) participants would share their data with medical professionals, 7 (32%) participants would share their data with scientists, and 2 (9%) participants would share their data with the producer of the product. A total of 7 (32%) participants explicitly mentioned not wanting to share their data with the producer of the product, 4 (18%) participants explicitly mentioned not wanting to share their data with the government, and 7 (32%) participants explicitly mentioned not wanting to share their data with the commercial sector:

My limit is the doctor who needs the data to help me.

P580

I can imagine health care professionals getting the data, that would not be so strange, but I still think I need to actively consent to transfer the data to them.

P588

If there is one group that I don’t trust it’s the government, unfortunately. If you look at the recent scandals...And they have computer systems that don’t work all that well...

P484

#### Barriers to and Requirements for the Use of Smart Toilets in the Home

Textbox 1 display an overview of the barriers to and requirements for the use of smart toilets in homes.

**Barriers to and requirements for the use of smart toilets.**

**Usability and everyday use**

• The smart toilet should fit the current design of regular toilets.

• The smart toilet should be sturdy and not be easily breakable.

• The toilet should be adjustable in height, as it is currently too high for some users

• The smart toilet should match the color and design of the regular toilet.

• The smart toilet should afford toilet habits, such as putting the seat up or closing the cover lid.

• The smart toilet should be easy to clean.

• The smart toilet should be inconspicuous so that privacy of use is warranted if desired.

**Data agency**

• The smart toilet should provide users with not only full access to their data but also the option to receive feedback only if there is a need for action or grave concern (*signaling*).

• The smart toilet should provide understandable and actionable feedback on relevant biomarkers and health data.

• The smart toilet should afford the option to share data or derived data with carers, general practitioners, or other medical professionals.

• The smart toilet should afford complete user control over further data sharing.

#### Sensitizers

Overall, 16 (52%) participants made remarks about the sensitizing questionnaires. A total of 11 (35%) participants mentioned the positive aspects of the questionnaires, mostly about the ease of use, whereas 14 (45%) participants mentioned negative aspects, mainly about questions they thought were irrelevant, such as those on the beauty of the prototype, or hard to answer, such as questions on mood and stress:

I liked the questionnaires, and they made me think about my role in the research. I started wondering about my data, and what [the researchers] would use them for. I did receive instructions beforehand, of course, that it would be about my experience and what using the smart toilet evoked in me, and those questions surely augmented that.

P843

Well, I thought the questionnaires were a bit dodgy. To me, the seat was the seat and nothing else, just like any seat. So I did not see the added value of the questions. There’s no feedback, so if I sit on my own toilet seat or yours, it’s all the same to me.

P850

There was a question about whether I thought the seat was beautiful, and I thought that made no sense. What is beautiful about a toilet seat? A white one or a brown one, it does not make a difference on how I sit. Well, I don’t really like the brown color, but it’s all part of the game.

P721

#### Saturation

Saturation was determined by calculating the number of unique themes for a base run of 4 transcripts and then establishing the percentage of new information coming forth from adding additional runs of 3 transcripts [51]. Top-level theme saturation was reached in the base run, with new top-level themes emerging below the 5% threshold for each additional run. Code saturation was achieved after including the first additional run, with new codes emerging below the 5% threshold for each additional run.

## Discussion

### Principal Findings

This study aimed to investigate how people experience using a smart toilet installed in their home: their perceived use cases for the innovation, the limitations and barriers to its everyday use, sharing the experiences with the toilet seat with others, and privacy and data sharing concerns. The results revealed that participants already found the current prototype quite usable, but most participants mentioned issues that can not only inform future iterations of the prototype but can also elucidate people’s expectations of smart bathroom technology. These expectations had a strong association with norms and behaviors around toilet use. The fact that the seat could not be raised, which entailed being seated when using the toilet, was problematic for many of the male participants and male visitors to the participating households. The fact that the current prototype was difficult to clean, especially because of the way it was connected to the toilet bowl, was mentioned by almost all participants, except for the 2 (4%) participants who admitted leaving toilet cleaning to their spouses. The fact that even for a prototype, the color and form play a role in acceptance shows that these aspects need to be considered when developing future iterations. On a more general level, this result shows that smart appliances need to fit everyday practices and norms.

Participants provided a broad range of use cases for the smart toilet seat. They saw signaling undetected health conditions or exacerbations of existing conditions as the most important potential application. Signaling occurs in the background, without notification or feedback, unless a result that warrants attention pops up. Further much-mentioned use cases were documenting all kinds of physiological and behavioral data to better understand oneself and using the toilet seat to diagnose certain conditions (which differs from signaling in that it is an active, overt process). Other use cases were personal science, in which the toilet seat is used to measure the effects of experiments with nutrition on participants’ health and using the toilet seat as a driver of behavioral change. These differ from existing frameworks in the literature [29,30], which lack signaling medical conditions but do cover use because of interest in the technology. The difference between these frameworks and the current results lies in the research sample. The cited studies included people interested in lived informatics and quantifying self-movement. Such people would be likely to actively adopt trackers, for instance, to measure their physical activity or heart rate variability. This study included a broader range of participants with a broader range of interests in technology per se and in the measurement of their own data. This broader range of interests is expressed in the number of participants who expressed worries about how feedback on physiological data may raise their stress levels or who do not see the added value in the smart seat. Further research can shed light on whether this sample better reflects the attitudes in the general population than the frameworks from lived informatics research.

The issue of data agency is a recurrent theme throughout the results of this study. When talking about their perceived use cases for the smart toilet seat, many participants expressed a desire for acting with and upon and learning from their data (eg, personal science use cases), whereas others expressed an opposite desire, that is, for data to be hidden from them unless there is something important that they need to act upon right away. This indeed shows that people must have the autonomy to determine the level of data availability by themselves for the technology to fit their needs. When talking about data ownership and privacy needs, the importance of data agency becomes even clearer. Data ownership and privacy protection are needs that must be met.

The many concerns participants expressed around sharing their experiences and their privacy needs confirm earlier research [31,52] and show that these issues should play a larger role in the development of smart home appliances. The study confirms work that shows that people have different needs when it comes to the on-stage, off-stage, or backstage use of technological innovations. Some participants were willing to present the smart seat to visitors and even go so far as to invite people into their homes to do so. Some were more reluctant and would discuss the smart seat only when the need to do so arose, and others, the backstage users, avoided sharing their experiences altogether, for instance, by “hiding” the smart seat in an upstairs bathroom. This study also shows that the same people can show different presentation preferences toward different people; what one shares with a close friend may differ from what one shares with a neighbor. The fact that this pattern is already present in a study with self-selection of participants (see subsequent paragraphs for the discussion of self-selection bias), in which we can expect more people who have no qualms about using or discussing toilets to participate, may very well mean that it is even more pronounced in the general population because people who avoid discussing this topic altogether could be less likely to take part in this research. This has consequences for the acceptability and design of smart appliances that are integrated into the home: it should be possible to put them away or hide them or their design should be inconspicuous.

Privacy and ownership of data in smart home appliances for health have been the focus of attention for at least a decade (eg, the study by Townsend et al [53]). The participants showed a strong preference for the ownership of their own data and having responsibility for sharing, transparency in who uses their data and for what purposes their data are used, and protection from undesired consequences. This reflects the findings of many other studies (eg, the studies by Kennedy et al* *[54], Forchuk et al [55], and Choi et al [56]). However, these concerns, as yet, have not been taken into account when developing actual products that enter the marketplace; very few of these products make the user the owner of their own data or provide them with the opportunity to control the flow of data and access. In future innovations, data management and privacy should play a more important role.

The study shows that the approach we followed, which consisted of sensitizing, provotyping, and discussion, was a successful method for supporting participants to voice their thoughts and concerns. The sensitizing phase succeeded in making people think about the smart toilet and various health subjects before the trial began. However, the participants’ responses also showed that sensitizing materials must be carefully designed. In this study, some participants showed irritation or other negative reactions because of questions they did not see the point of, such as the questions on esthetic aspects, part of the user experience questionnaire covering hedonic aspects. Some participants did not see the relevance of answering questions about the “beauty” of a prototype, as it was obviously not the finished product. Interestingly, their irritation did make them consider and talk about esthetics, a facet of the prototype design that they would otherwise never have thought of. However, to ensure that participants are not alienated by the sensitizing materials, these materials should be better pilot-tested and more carefully worded. Moreover, the burden of the sensitizing phase should be as low as possible.

In this study, provotyping proved very successful. First and foremost, it gave participants the necessary experiences for talking about barriers and needs surrounding smart appliances used in sensitive areas of the home. Moreover, the approach succeeded in making the normally unsaid factors available for discussion: norms, taboos, and cultural practices that are so embedded in everyday life that they escape conscious scrutiny. The most important example in this study were the conversations on participants’ toilet use habits, which they would normally never talk about. These conversations presented valuable insights that may even go beyond the current research setting: there is surprisingly little, if any, literature on the everyday practices of toilet use; the current literature only mentions toilet use when practices are greatly different from the Western norms and standards, such as works on communal toilet facilities in South Africa [57] and East and Southeast Asia [58,59] or on latrine use in rural India [60]. A second source of literature on toilet use is a side note in a work on ensuring sustainability through water use reduction; here, toilet use is mentioned as being “highly routinised” and therefore “very difficult to change” [61,62]. The current results are, therefore, also interesting as an ethnography of toilet use practices, especially when it comes to aspects of toilet use that are so embedded in everyday life that they usually remain unsaid in scientific discourse: standing up while urinating, lifting and lowering of toilet seats, and hygiene aspects.

The discussion phase served its purpose and delivered a rich qualitative data set. Unfortunately, it proved impossible to have all participants join focus groups of 3 to 4 people to obtain the desired group size. The number of participants who ended their provotyping phase in roughly the same time frame was limited by the number of toilet seats we had at our disposal; moreover, the COVID-19 pandemic and limited availability of many participants also played a role. Taking part in smaller groups means that although every participant gets ample time to share their thoughts, they have less opportunity to get inspired by what others say and hear different voices. This limitation may have reduced the richness and value of the data in this research.

Although we did our best to eliminate bias, no research can escape potential influences on validity. The sample we included in this study is likely to have a certain amount of participant bias because of self-selection, which is, for example, visible in the age of the participants. Even though we aimed to include people from all age groups older than 18 years in similar numbers, we did not manage to achieve that; the average age of the participants was 62 (SD 13.97; median 68; range 28-84) years, and more than half (25/48, 52%) of the participants were aged between 60 and 84 years. This may have affected the results because the results now mostly reflect the viewpoints of older people interested in taking part in this kind of research. However, the age of the participants could also be seen as an indication of potential interest, with older users being naturally more inclined to be concerned about their health in general and their gut health in particular. Furthermore, the ATI scores of the participant group resemble those of a comparable general population, which means that the participants are likely to be representative of a broader audience when it comes to interest in technology use in everyday life. Finally, the self-selection bias could mean that the reservations participants mentioned about sharing experiences regarding the smart toilet and about data ownership and privacy could very well be more pronounced in the general population, as people who have very strong reservations are unlikely to take part in this kind of research.

A self-serving researcher bias may have arisen from the aims of the research program. Members of the research team were deeply involved in the development of the smart toilet, which may have curbed the participants’ inclination to express negative opinions about the seat. However, the main interviewers (SH and MB) had no such vested interests in the success of this prototype; moreover, the results show that the participants felt free to cast their doubts, saying that they have no use for the smart toilet or feel skeptical about its efficacy.

Third, as stated before, because the current scientific literature on toilet habits and use is lacking, it is difficult to evaluate the generalizability of the results of this research. Owing to logistic limitations, all participants came from the province of Gelderland in the Netherlands and its neighboring regions, so the results found in this study might theoretically be limited to this region. However, when it comes to the current toilet design and use, this region can be seen as representative of large swathes of the global population. In the European Union, 98% of the population has similar toilets [63], and so does the US population [64]. The results from this study almost certainly would not apply to many people in Asia, for instance, those in China and India, where squat toilets are ubiquitous. Further research can elucidate whether our hypothesis that the current results are valid for those parts of the world that have similar-style toilets is correct.

The smart toilet described in this research is not unique. There are a number of similar initiatives around the world, both in academia and industry, such as the Stanford smart toilet [65], Toto smart toilet [66], and Rochester Institute of Technology smart toilet [67], and there are modules that can be placed inside regular toilets to measure urine contents, such as the Withings U-Scan (see the description in the study by Sequeira-Antunes and Ferreira [68]). However, research on and toward these toilets and modules has as yet concentrated only on technical efficacy. To our knowledge, there has not been any research on use cases as seen by potential users; the barriers to and facilitators of acceptance; and other issues of use in general daily life, such as fit with culture and habits. Our study not only sheds light on our own prototype in these regards but can also inform the design and development of other endeavors in the field.

Finally, this study concentrated on the use cases, needs, and barriers put forward by potential end users. However, the acceptance and efficacy of smart innovations in health care depend on many more stakeholders, including health care professionals, social workers, health insurance providers, and public policy makers. Further research will include their voices as well.

### Conclusions

This study showed that participants felt that a smart toilet seat could be acceptable and effective, as long as it fits everyday practices concerning toilet use and hygiene. The use cases they envisioned ranged from signaling the deterioration of health conditions to documenting health data to informing diagnoses to engaging in personal science endeavors to driving behavioral change. Participants differed in how much they wanted to share their use of the smart toilet with others; whereas the majority (17/31, 55%) shared their experiences of using the toilet with others, 4 (13%) participants never talked about the toilet with others or let others see or use the toilet, and 10 (32%) participants shared their experiences with some people but not with others. When it comes to the data produced by the smart toilet seat, participants expressed a need for ownership, transparency, and control; most participants (18/31, 58%), however, would share their data with health care professionals. Finally, the method used in this study proved to be a successful way to support people in talking about aspects of their behavior and everyday life that normally remain unspoken.

The results of the study not only inform further iterations of the smart toilet prototype and the smart bathroom program but also have relevance outside these applications. The categories of use cases mentioned by the participants differ from those in the current literature and may provide a better reflection of average users than the categories of use cases mentioned in studies from the realm of quantified self-movement. Using or avoiding the use of technology for self-presentation is a relatively underresearched topic, which may, however, have a great impact on the acceptance and public use of smart appliances, wearable technology, and other technologies for supporting people’s health. Future research on these subjects can further strengthen our knowledge.

## Appendix

Multimedia Appendix 1 Sensitizing questionnaires.

## Abbreviations

ATI: Affinity for Technology Interaction
EMA: ecologic momentary assessment
SUS: Systemic Usability Scale

## Data Availability

The data sets used or analyzed during this study are available from the corresponding author upon reasonable request.
