# Supplementary material for: Perceived Use Cases, Barriers, and Requirements for a Smart Health-Tracking Toilet Seat: Qualitative Focus Group Study
Source: JMIR Hum Factors. 2023 Aug 11;10:e44850. doi: 10.2196/44850 (PMC10457698; doi:10.2196/44850)
Supplement: Multimedia Appendix 1 [file humanfactors_v10i1e44850_app1.docx]

Appendix I – Sensitizing Questionnaires

1) Intake questionnaire

a. General

1. Age in years.

2. Length in cm

3. Weight in kg

4. Sex: female/male/other/don't want to say

5. Overall feeling of health: scale 1–7

6. Are you currently suffering from Covid-19? Yes/no/unknown

7. Do you have any chronic conditions?

a. None

b. Diabetes I/II

c. Heart disease

d. Asthma, COPD

e. Arthrosis

f. Other, namely:

b. Mental Wellbeing Questionnaire

Last week....

1. … I've been feeling optimistic about the future

2. … I've been feeling useful

3. … I’ve been feeling relaxed

4. … I've been feeling interested in other people

5. … I've been feeling challenged

6. … It has been easy for me to be satisfied with my living conditions.

7. … I've been able to live a worthy life

8. … I've had energy to spare

9. … I've been dealing with problems well

10. … I've been thinking clearly

11. … I've been feeling good about myself

12. … I've been feeling stressed

13. … I've been feeling close to other people

14. … I've been feeling confident

15. … I've been able to make up my own mind about things

16. … I've been feeling loved

17. … I've been interested in new things

18. … I've been feeling cheerful

19. … I've been feeling like my life was useful

20. … I've been feeling like others (partner, family, friends) helped me deal with daily life

21. … I've been feeling bored

Answers on a scale of 1-7 ranging from ‘none of the time’ to ‘all of the time’.

c. Perceived Stress Scale

1. How often have you been upset because of something that happened unexpectedly?

2. How often have you been feeling unable to control the important things in your life?

3. How often have you been feeling nervous and ‘stressed’?

4. How often have you been feeling confident about your ability to handle your personal problems?

5. How often have you been feeling that things were going your way?

6. How often have you been feeling that you could not cope with all the things that you had to do?

7. How often have you been able to control irritations in your life?

8. How often have you been feeling that you were on top of things?

9. How often have you been angered because of things that were outside of your control?

10. How often have you been feeling like difficulties were piling up so high that you could not overcome them?

Answer on a scale of 1-4 ranging from ‘never’ to ‘very often'.

d. Digestive health questionnaire

1. Have you experienced digestive discomfort such as your abdomen feels heavy, bloating, stomachache, altered bowel habit, flatulence or other abdominal discomfort?

2. How often have you had discomfort or pain anywhere in your abdomen?

3. Does this discomfort/pain get better, or stop after you have had a bowel movement?

4. When this discomfort or pain began, did you have more frequent bowel movements?

5. When this discomfort or pain began, did you have hard or lumpy stools?

6. How often has your abdominal discomfort/pain prevented you from sleeping, or woken you during the night?

7. How often do you feel the need to strain to pass a motion (stool)?

8. How often do you feel that your bowel has not been emptied after a bowel movement?

9. How often have you been troubled with loose, mushy or watery stools?

10. How often do you experience abdominal bloating?

11. How often do you feel uncomfortably full after a regular-sized meal?

12. How often do you experience abdominal distension?

Answers on a scale of 1-7 ranging from ‘never’ to ‘always’.

e. Open question regarding the smart toilet seat:

1. What do you expect from the smart toilet? (Open text entry)

2. What would you want to achieve with or learn from the smart toilet? (Open text entry)

3. How important is this goal for you? (Scale 1–7, totally unimportant to extremely important)

4. How detrimental would it be for you if you cannot achieve this goal? (Scale 1–7, totally detrimental to no consequences at all)

5. What would change if you achieved this goal? And what would change if you would not? (Open text entry)

6. Do you think achieving the goal has disadvantages as well? (less fun, less easy, less cozy, etcetera?) (Open text entry)

7. Do you expect using the smart toilet to be difficult, easy to learn, time-consuming, worth it? (Open text entry)

8. If you could equip your house with a smart toilet (so not for research), would you? Why? (Open text entry)

2) Installation questionnaire

1. Installation of seat

a. How easy? Scale 1-7 ranging from ‘extremely complicated’ to ‘extremely simple’.

b. What should be easier?

2. First impression of seat usage

a. How easy? Scale 1-7 ranging from ‘extremely complicated’ to ‘extremely simple’.

b. What should be easier?

3. Did the first impressions of installing the seat match your expectations? What did match and what did not?

3) User experience questionnaire

a. User experience

1. Installation and deinstallation of seat

a. Does it happen you want to remove the seat for a while? Yes/no. If yes: why?

b. How easy? Scale 1-7 ranging from ‘extremely complicated’ to ‘extremely simple’

c. What should be easier?

2. Cleaning of seat

a. How easy? Scale 1-7 ranging from ‘extremely complicated’ to ‘extremely simple’

b. What should be easier?

3. Impression of seat usage

a. How easy? Scale 1-7 ranging from ‘extremely complicated’ to ‘extremely simple’

b. What should be easier?

c. Stability of the seat? Scale 1-7 ranging from ‘extremely instable’ to ‘extremely stable’

d. Height of the seat? Too low/low/good/high/too high

e. Is there any discomfort during the use of the seat? Yes/no. If yes: what?

f. Do you get used to the seat? Scale 1-7 ranging from ‘still not used to’ to ‘used to them very quickly’

b. Systems Usability Scale

1. I think I want to use the smart toilet often.

2. I think the smart toilet is unnecessarily complex.

3. I think the smart toilet is user-friendly.

4. I think I need the support of a technical person to use the smart toilet.

5. I think the different functions in the smart toilet are well integrated.

6. I think there is too much inconsistency in the smart toilet.

7. I imagine that most people would learn to use the smart toilet very quickly.

8. I find the smart toilet very cumbersome to use.

9. I feel very confident in using the smart toilet.

10. I had to learn many things before I could get started with the smart toilet

c. Hedonic quality of usability (scale 1–7 strongly disagree to strongly agree)

1. Is the smart toilet: fun / modern / intrusive / funny / embarrassing / annoying / fulfilling needs / robust / beautiful?

d. Pragmatic quality of usability (scale 1–7 strongly disagree to strongly agree)

1. Is the smart toilet: exact / detailed / clear / credible / useful?

e. Perceived efficacy (scale 1–7 strongly disagree to strongly agree)

1. The smart toilet:

a. ...could help me achieve my goals (when score is above 4: what goal? How can the smart toilet help?)

b. ...could make it easier to achieve my goals (when score is above 4: what goal? How can the smart toilet help?)

c. ...makes my life harder (when score is above 4: in what way?)

f. Affinity with Technological Interaction scale, Dutch version (ATI; all answers on a scale from 1 (do not agree at all) to 6 (fully agree).

1. I like to occupy myself in greater detail with technical systems.

2. I like testing the functions of new technical systems.

3. I predominantly deal with technical systems because I have to.

4. When I have a new technical system in front of me, I try it out intensively.

5. I enjoy spending time becoming acquainted with a new technical system.

6. It is enough for me that a technical system works; I don’t care how or why.

7. I try to understand how a technical system exactly works.

8. It is enough for me to know the basic functions of a technical system.

9. I try to make full use of the capabilities of a technical system
